# Nitro Fatty Acids (NO_2_-FAs): An Emerging Class of Bioactive Fatty Acids

**DOI:** 10.3390/molecules26247536

**Published:** 2021-12-13

**Authors:** Giorgos S. Koutoulogenis, George Kokotos

**Affiliations:** 1 Department of Chemistry, National and Kapodistrian University of Athens, Panepistimiopolis, 15771 Athens, Greece; gkoutoul@chem.uoa.gr; 2 Center of Excellence for Drug Design and Discovery, National and Kapodistrian University of Athens, 15771 Athens, Greece

**Keywords:** cancer, inflammation, nitroalkenes, nitro fatty acids, NF-κB, synthesis

## Abstract

Unsaturated nitro fatty acids (NO_2_-FAs) constitute a category of molecules that may be formed endogenously by the reaction of unsaturated fatty acids (UFAs) with secondary species of nitrogen monoxide and nitrite anions. The warhead of NO_2_-FAs is a nitroalkene moiety, which is a potent Michael acceptor and can undergo nucleophilic attack from thiol groups of biologically relevant proteins, showcasing the value of these molecules regarding their therapeutic potential against many diseases. In general, NO_2_-FAs inhibit nuclear factorκ-B (NF-κB), and simultaneously they activate nuclear factor (erythroid derived)-like 2 (Nrf2), which activates an antioxidant signaling pathway. NO_2_-FAs can be synthesized not only endogenously in the organism, but in a synthetic laboratory as well, either by a step-by-step synthesis or by a direct nitration of UFAs. The step-by-step synthesis requires specific precursor compounds and is in position to afford the desired NO_2_-FAs with a certain position of the nitro group. On the contrary, the direct nitration of UFAs is not a selective methodology; thus, it affords a mixture of all possible nitro isomers.

## 1. Introduction

**Scheme 1:**
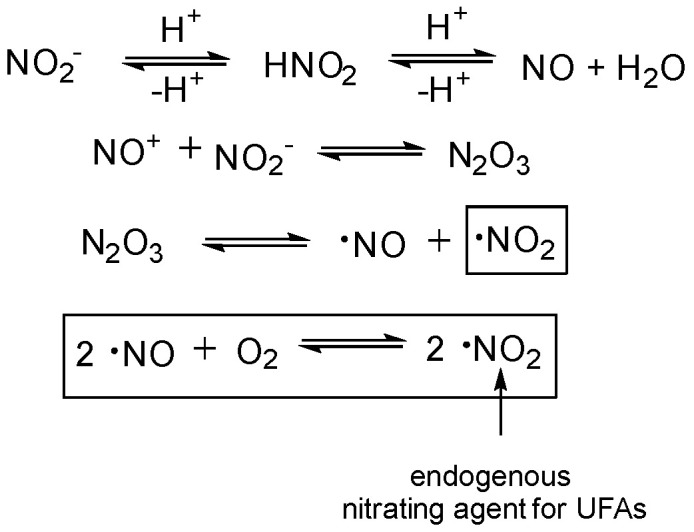
Endogenous formation of nitrogen dioxide, the reactive nitrating agent of UFAs.

Nitro fatty acids (NO_2_-FAs) is a class of compounds that are endogenously formed by the reaction of unsaturated fatty acids (UFAs) [oleic (OA), linoleic (LA), etc.] with secondary products of nitrogen monoxide (NO**^.^**) and nitrite anions (NO_2_^−^) (Scheme 1), resulting in the formation of a reactive nitroalkene moiety [1,2,3]. These nitrated lipids have been detected and characterized in several tissues and biological fluids employing mass spectrometry-based approaches [4,5,6,7]. A recent review article summarizes the applications of such approaches to identify NO_2_-FAs and their protein adducts [8]. A very recent study defines the urine nitrolipidome, highlighting the presence of nitro derivatives of conjugated linoleic (cLA) and linolenic acid (cLnA) [9].

**Scheme 2:**
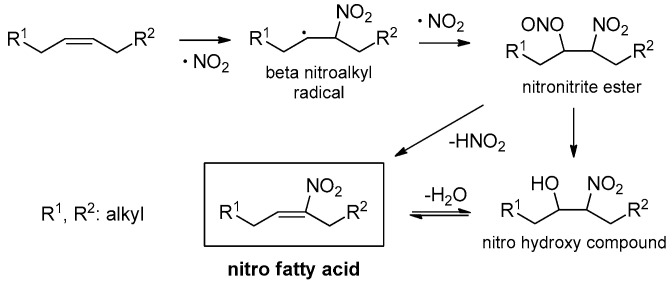
Endogenous nitration of UFAs [3].

The endogenous formation of NO_2_-FAs begins with the formation of a β-nitroalkyl radical and its reaction to a nitronitrite or a nitrohydroxy intermediate (Scheme 2). The formation of NO_2_-FAs is achieved, when an elimination of nitrous acid of the nitronitrite ester or a dehydration of the nitrohydroxy compound takes place, respectively [3]. NO_2_-FAs have at least one nitrated double bond, granting in this way an electrophilic nature to the molecule and for that reason NO_2_-FAs can react with thiol groups very fast to generate reversible Michael addition products, due to the high reactivity of the electrophilic warhead of these molecules [10,11,12]. To clarify the way the cells signal with NO_2_-FAs and in order to better understand their pharmaceutical benefits, their biophysical properties and behavior in water have been studied by Vacek et al. [13].

**Figure 1:**
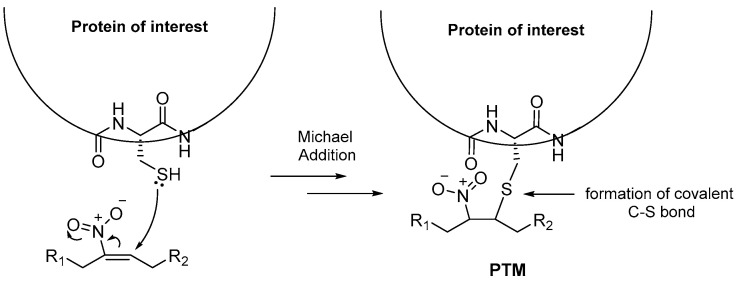
Post-translational modification of protein of interest by Michael addition [3].

NO_2_-FAs have been reported to form covalent adducts with cysteine residues of Kelch-like ECH-associated protein (Keap-1), resulting in activation of nuclear factor erythroid 2-related factor 2 (Nrf2). This activation of Nrf2 induces the expression of genes responsible for an overall antioxidant and cell-protecting function [14,15,16]. The covalent interactions between NO_2_-FAs and the Cys residues are reported to induce anti-inflammatory cell signaling as well [3]. The formation of covalent adducts between NO_2_-FAs and the protein of interest, via the formation of a new C-S bond, causes the protein to change its overall structure, therefore its function as well. This structural change of the protein is called Post-Translational Modification (PTM) (Figure 1) [3]. Understanding the way NO_2_-FAs interact with specific residues of the catalytic site of several proteins, residues beyond the catalytic pocket or even non-enzymatic proteins (such as structural proteins or transcriptional factors) each time, is of paramount importance for the Structure-Activity Relationship (SAR), in order to maximize the therapeutic benefits of NO_2_-FAs and minimize the harmful effects of the diseases.

NO_2_-FAs are also reported for alkylating important Cys residues of p65 and p50 subunits of nuclear factor κB (NF-κB), preventing the endogenous formation of a variety of pro-inflammatory mediators, responsible for diseases, like vascular inflammation and pro-fibrotic responses [17,18,19].

Besides the ability of the organism to synthesize by itself NO_2_-FAs during a state of inflammation, NO_2_-FAs can be synthesized in the laboratory. These nitroalkene-bearing compounds can be synthesized either by a direct nitration of the double bond of the corresponding UFA, where in that case an approximately 1:1 ratio of nitro regio-isomers are formed, or by a step-by-step synthesis. The step-by-step approach involves a Henry reaction between the appropriate aldehyde and the nitro component, depending on the desired nitration position. The final step of this synthetic pathway is an E_2_ elimination for the formation of the desired nitroalkene moiety, followed by an ester deprotection step. The various synthetic methods developed so far will be discussed extensively, along with the pros and cons of each method.

## 2. Bioactivities of NO_2_-FAs

### 2.1. Interaction of Cysteine Residues with NO_2_-FAs

Cysteine residues of several proteins may react with numerous electrophilic compounds, due to the high nucleophilic reactivity of the -SH group, in order to form covalent adducts via a Michael addition. The environment where the Cys residue is located plays a predominant role to define the pKa value of the -SH group, which for the residue Cys in aqueous solution at pH 7.2 is 8.33 [20]. The acidity of the -SH proton increases significantly when next to the Cys residue exist basic residues like His, Lys and Arg, metal centers from different enzymes (e.g. the iron metal center of hemoglobin), and different aromatic amino acids, such as Tyr and Trp (pi interaction with the thiol group) [20]. The above factors have as a result the formation of the highly reactive thiolate anion (RS^−^), which is a much better nucleophile in comparison with the free thiol group of the Cys residue to undergo oxidative or Michael reactions [21,22].

The covalent formation of adducts between several Cys residues and NO_2_-FAs play a pivotal role to many biochemical pathways with beneficial outcome for the organism. NO_2_-FAs exhibit potent anti-inflammatory effects in animal models of inflammation [16,17,18]. These electrophilic molecules interact with specific Cys residues of many regulatory proteins, like Keap-1, Nrf2, NF-κB, Peroxisome Proliferator-Activated Receptor γ (PPARγ), Heat Shock Proteins (HSP), 5-Lipoxygenase (5-LO) and others in order to regulate many diseases.

### 2.2. 10-Nitro Oleic Acid (CXA-10) and Its Derivatives as Anti-Inflammatory and Cytotoxic Agents

**Figure 2:**
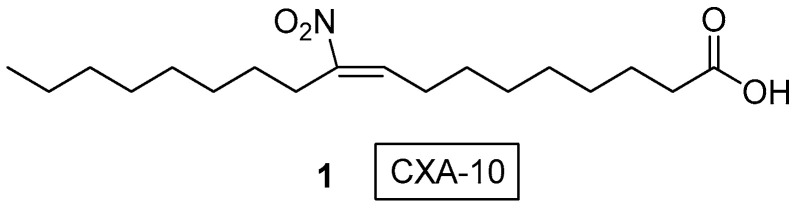
CXA-10 at ongoing phase II clinical trials as an investigational drug against PAH, FSGS and asthma.

One of the most studied NO_2_-FAs is CXA-10 (**1**) (10-nitro oleic acid, 10-NO_2_-OA), which has entered Phase II clinical trial against Pulmonary Arterial Hypertension (PAH), Focal Segmental Glomerulosclerosis (FSGS) and asthma, by Complexa Inc., despite that it was first studied against acute kidney injury [23,24,25]. CXA-10 is the regio-isomer where the nitro group is located only at C10 of OA (Figure 2) exhibiting unique biological activity. Some of the pharmacokinetic (PK) and pharmacodynamic (PD) effects of oral CXA-10 in healthy and obese subjects were described by Jorkasky, et al. in 2019 [26].

NO_2_-FAs exhibit potent therapeutic properties in models of PAH caused by hypoxia or obesity [27,28]. More specifically, lung macrophage infiltration, expression of pro-inflammatory cytokines and glucose tolerance were improved significantly when NO_2_-OA was administrated to a PAH murine model. Characteristic lesions of PAH, like ventricular hypertrophy, fibrosis, etc., dropped significantly, unlike some biological markers like Monocyte Chemotactic Protein-1 (MCP-1), Interleukin-6 (IL-6), Tumor Necrosis Factor α (TNFα) and some macrophage markers (F4/80, CD68) levels in blood of High-Fat Diet (HFD) mice were increased. These HFD data showed that inflammation dropped significantly when 10-NO_2_-OA was administered, demonstrating once again the anti-inflammatory potency of 10-NO_2_-OA. NO_2_-FAs have been reported, as well to inhibit significantly the inflammation caused by bleomycin-mediated lung injury in mice by inhibiting Intratracheal Administration of Bleomycin (ITB)-induced pro-inflammatory mediators [29].

Neumann et al. have reported that CXA-10 increases significantly the antiproliferative effects of some known antineoplastic DNA-damaging agents, such as olaparib, cisplatin, doxorubicin against triple-negative breast cancer (TNBC) [30]. As a result, TNBC epithelial cell growth drops significantly by inhibiting the NF-κB signaling pathway. This signaling inhibition by CXA-10 happens due to the PTM effect on Cys residues to NF-κB subunit kinase β (IΚΚβ) and the NF-κB Re-1a protein, preventing this way DNA binding and an upcoming Re-1 protein-induced degradation of proteins. Moreover, CXA-10 inhibited TNFα-induced NF-κB activity, having as a result the suppression of harmful target genes, responsible for the expression of metastasis-related proteins like Intercellular Adhesion Molecule-1 (ICAM-1) and urokinase-type plasminogen activator [31].

**Figure 3:**
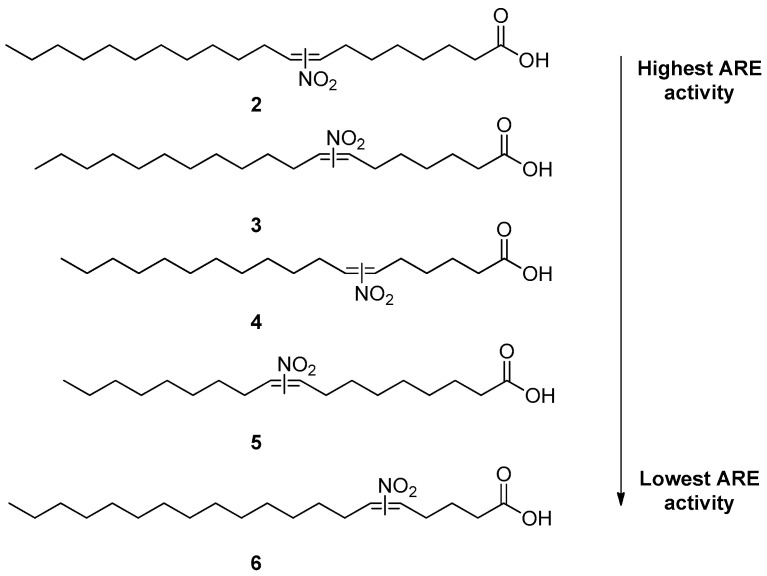
Activation rate of ARE related to different NO_2_-FAs.

**Figure 4:**
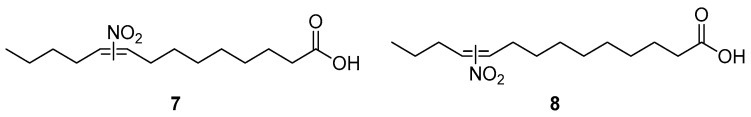
Nitroalkenes showing similar deactivation rate of TNFα-induced NF-κB signaling pathway with NO_2_-OA.

A study in 2018 by Freeman and coworkers reported twenty new nitroalkenes as candidates for the activation of Antioxidant Response Element (ARE), which means activation of Nrf2 and inhibition of the NF-κB signaling pathway [32]. For better understanding of the importance of the nitroalkene position or the carbon chain length of the tested nitroalkenes, a variety of nitroalkenes was synthesized. That work aimed to clarify if the synthetic nitroalkenes activate ARE luciferase or inhibit TNF-α induced NF-κB signaling pathway. The importance ofthe terminal or interior position of the nitroalkene was examined as well. The nitroalkenes with the highest ARE activity are shown below, ranked from highest to lowest activity (Figure 3). It is noted that three out of five nitroalkenes have an ω-12 nitration position. Regarding the NF-κB inhibition, two nitroalkenes showed the same inhibition rate with NO_2_-OA and are shown below (Figure 4).

**Figure 5:**
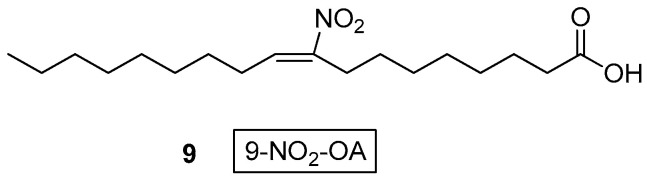
9-NO_2_-OA as a cytotoxic agent against colorectal cancer cell lines.

In 2018, Kühn et al. reported the other regio-isomer of CXA-10, 9-nitro oleic acid (9-NO_2_-OA) (**9**) (Figure 5) as a cytotoxic agent against colorectal cancer cell lines HCT-116 and HT-29. The cell viability of these cancer cell lines was suppressed significantly by 9-NO_2_-OA through a caspase-dependent apoptosis, via the intrisic apoptotic pathway [33]. This effect against cancer cell lines is based on the antioxidant effects of NO_2_-FAs and the capability of 9-NO_2_-OA to target mitochondria and deregulate their membrane and respiration, having as a result the final apoptosis of the cells. The entire cytotoxic effect is based on the Complex II inhibition by 9-NO_2_-OA, which is reversible and pH-dependent. As the pH gets more basic, the R-SH of the Cys residue deprotonates to the more reactive thiolate R-S^−^, giving this way a higher inhibition rate by 9-NO_2_-OA, since Michael additions are favored under basic conditions.

In order to understand whether the Michael acceptor is enough for the tested molecules to demonstrate cytotoxic effects against cancer cell lines, the combination of monomethyl fumarate (MMF) and 15-deoxy-delta-12,14-prostaglandin J_2_ (PGJ_2_) with HCT-116 cells was studied and compared with 9-NO_2_-OA’s cytotoxic effect [33]. The cell line was treated with all the three candidates for 48 h and the best results came from 9-NO_2_-OA, since it showed the highest cytotoxicity of all (IC_50_ 6.5 μM), followed by PGJ_2_ (IC_50_17.6 μM), when MMF presented no cytotoxicity at all.

Most recently, fifteen NO_2_-FAs have been studied for their effects on activation of Nrf2, and inhibition of NF-κB signaling pathway, cyclooxygenase-2 (COX-2), LO and soluble Epoxide Hydrolase (sEH), as well as for their cytotoxic activity on colorectal cancer cells [34]. All the new NO_2_-FAs were compared with the lead compound 9-NO_2_-OA (**9**) (Figure 5).

**Figure 6:**
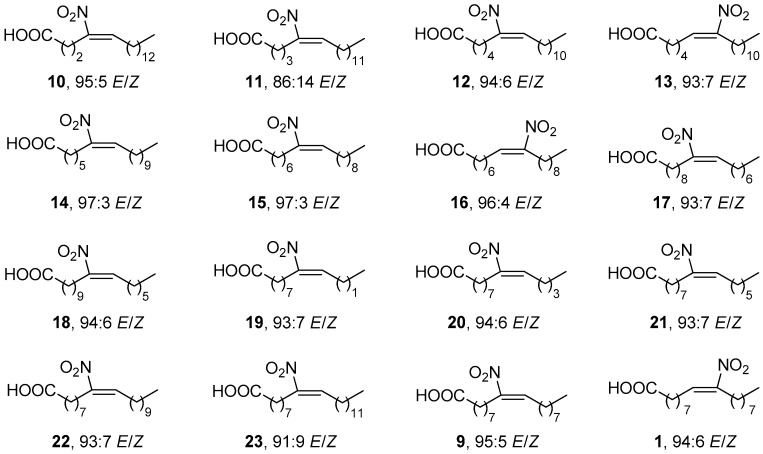
New NO_2_-FAs for SAR studies.

The structures of NO_2_-FAs included in this SAR study are shown in Figure 6. The carbon chain length varies between twelve to twenty-two total carbon atoms and the nitro group is inserted from position C4 to C11 each time, close or away from the carboxylic group.

The inhibitory activity of these NO_2_-FAs against the NF-κB signaling pathway (p65 and p50) in colorectal cancer cells was studied in comparison with 9-NO_2_-OA [34]. The cytotoxic effect of the tested NO_2_-FAs against subunit p65 showed that compounds **22** and **23** demonstrated slightly worse activity than 9-NO_2_-OA. Compounds **14**, **15** and **16** exhibited a stronger cytotoxicity than compound **9**, with compound **14** being the most potent agent. The same results were demonstrated against subunit p50, where the only difference was the fact that compounds **14**, **15** and **16** exhibited a slightly lower potency.

All of the tested NO_2_-FAs induced Nrf2, as it was demonstrated by Western blot and ELISA [34]. The majority of the tested compounds with a carbon chain length greater than sixteen, led to a significant Nrf2 gene expression. Also, NO_2_-FAs where the nitroalkene moiety position was greater than six, led to a 3-fold induction of Nrf2. As a negative experiment control, OA was ascertained that did not affect the Nrf2 gene expression at all.

The SAR study against LOs showed that compound **23** exhibited significant inhibition of 5-LO with an IC_50_ value of 0.3 ± 1.2 μM, while alternating the nitroalkene moiety position did not cause any significant result against 5-LO [34]. Compound **10** exhibited more potent inhibition of 5-LO (IC_50_ 0.4 ± 1.2 μM) in comparison to 9-NO_2_-OA (**9**) (IC_50_ 1.1 ± 1.2 μΜ). 12-LO was not inhibited at all, while 15-LO showed a weaker inhibition effect in comparison to 5-LO. The tested NO_2_-FAs showed an average inhibition of sEH and failed to inhibit COX-2 [34].

Freeman, et al. reported that invivo administration of NO_2_-OA at nanomolar levels led to the inhibition of atherosclerotic lesion formation and promotion of plaque stability to mice suffering from atherosclerosis [35]. Many antiatherogenic and cardioprotective ω-3 PUFAs [36], that can be endogenously nitrated by the reactive species of nitrogen monoxide, as described before, can explain the beneficial results of these ω-3 fatty acids against cardiovascular diseases. Human atherosclerosis is a typical vessel-derived disease, so NO_2_-FAs could be an alternative therapy in that case. NO_2_-OA has also been reported by Braumann et al. that can attenuate myocardial fibrosis in a murine model of dilated cardiomyopathy (DCM) [37].

Several reports state that NO_2_-OA might exert a beneficial and protective role against cardiovascular diseases [38,39,40]. Lipoprotein-associated phospholipase A_2_ (Lp-PLA_2_) is a cardiovascular risk biomarker, due to its link with coronary disease and stroke [41,42]. The way NO_2_-OA decreases the levels of Lp-PLA_2_ biomarker in blood can be explained by the simultaneous downregulation of cytokines, causing inflammation and the enchancement of SuperOxide Dismutase (SOD) activity. NO_2_-OA may be an alternative as a therapeutic compound for encountering Ventricular Tachykardia (VT), since a murine model demonstrated significantly reduced chance of appearing VT, after NO_2_-OA was administrated [43].

The induction of resistance to ischemic cardiac injury by NO_2_-FAs via the modulation of mitochondrial respiration at Complex II has been reported in 2016 by Schopfer et al. [44]. More specifically, NO_2_-OA inhibited in a reversible manner the respiration function of mitochondria cells in rat heart via interactions with Complex II, resulting in downregulation of the superoxide formation.

Another very important metabolic pathway for the regulation of inflammation is induced by cycloxygenase-2 (COX-2)-derived prostaglandins (PGs) and by the synthesis of leukotrienes (LTs), which are dependent on LO [45]. Some eicosanoids, like LTB_4_ and PGE_2_, modulate very strongly the activation of leukocytes, a type of cells of the immune system responsible for protecting the organism against infectious diseases and foreign microorganisms [46,47]. In order LTs to be synthesized, arachidonic acid (AA) must be hydrolysed from the *sn*-2 position of the membrane phospholipids by phospholipase A_2_ (PLA_2_), which is then oxidized by 5-LO giving rise to the synthesis of lipid mediators including LTs, PGs and lysophosphatidic acid [48,49]. 5-LO was found to be inhibited by the interaction of some electrophilic lipids with some important catalytic amino acid residues [50], so Awaad et al. studied if NO_2_-FAs can interact with 5-LO [51]. The PTM mechanism of NO_2_-OA against 5-LO was clarified with extending studies, including proteomics and mutagenesis. When 5-LO was treated with NO_2_-OA, covalent adducts between the nitroalkene and different histidine residues (125, 360, 362, 367 and 432) were reported. These residues did not show any adduct formation with OA. These His residues, forming adducts with NO_2_-OA, do not exist only in 5-LO, but they exist in 12-LO and 15-LO as well, which means NO_2_-FAs cannot be considered as selective inhibitors over 5-LO. Some Cys residues (159, 300, 416 and 418) consist of a building unit found only in 5-LO enzyme and are very important for the enzyme’s function [52]. A peptide chain of 5-LO, containing Cys 416 and 418, was alkylated by NO_2_-OA, resulting in an overall enzyme inhibition. In that study, nitro linoleic acid (NO_2_-LA) was examined as well to check its inhibitory potency against recombinant 5-LO along with NO_2_-OA. The IC_50_s of these two compounds against recombinant 5-LO were 0.3 ± 0.06 μM and 0.004 ± 0.002 μM for NO_2_-OA and NO_2_-LA, respectively. These IC_50_ values show a better inhibition of NO_2_-LA over NO_2_-OA in the case of recombinant 5-LO.

In 2016, Maucher et al. reported the use of CXA-10 against 5-LO [53], as Awwad did in 2014 [51]. They tested many electrophilic species against 5-LO, including mainly phenols, quinones, nitroalkenes and other electrophilic lipids [53]. They showed that CXA-10, as a potent Michael acceptor, has the capability of forming covalent adducts with the surface residues Cys416 and 418, modulating this way the structure of 5-LO. This structure modulation has the result of 5-LO inhibition and the prevention of the oxidation of AA and its upcoming inflammation storm.

### 2.3. PPARγ-Regulated Diseases Attenuated by NO_2_-FAs

PPARs consist of a group of nuclear receptor proteins whose role is the regulation of the expression of genes [54]. More specific, their regulating role is crucial for development, metabolism, cellular differentiation and tumorigenesis [55,56,57,58]. PPARγ is one of the four nuclear receptor proteins (PPARα, β/δ, γ) and is responsible for the fatty acid storage and the metabolism of glucose [59], the regulation of the differentiation of adipocyte [60], the increase of insulin sensitivity and the M2 macrophage activation in mice [61,62].

NO_2_-FAs may act as partial agonists of PPARγ [62,63,64]. PPARγ unit has the Cys285 residue that forms a covalent adduct with NO_2_-FAs, explaining the interaction between PPARγ and NO_2_-FAs. 9- and 10-NO_2_-OA are potent and selective activating ligands of PPARγ at physiological concentrations (100 nM), while PPARα and PPARδ require higher concentration levels of NO_2_-OA (~300 nM) to be activated. NO_2_-OA regio-isomers are reported to be responsible for the induction of PPARγ-dependent adipogenesis and the uptake of deoxyglucose in preadipocytes.

As stated before, NO_2_-FAs regulate some very important functionalities of the organism like metabolism of glucose, insulin sensitivity, regulation of adipocyte tissue and M2 macrophage activation [59,60,61,62]. For example, partial agonists NO_2_-OA and NO_2_-LA activate PPARγ and then CD36 protein expression is regulated, causing the M2 macrophage activation. Insulin sensitivity is increased by the upregulation of PPARγ-dependent expression of glucose transporter type 4 (GLUT4) and coactivator 1-alpha by the aforementioned NO_2_-FAs. The expression of GLUT4 and coactivator 1-alpha is responsible for the reduction of fat accumulation, pro-inflammatory cytokines and for the increase of mitochondrial mass. These are some beneficial properties of NO_2_-FAs which, as stated before, consist of partial agonists of PPARγ and not full agonists like rosiglitazone and pioglitazone. Rosiglitazone and pioglitazone are thiazolidinedione (TZD) drugs, which, during their administration, cause adverse side-effects like edema, adipogenesis and gaining fat, which are not caused when NO_2_-FAs are administrated in mice [40,65].

In 2010, Schopfer et al. showed that NO_2_-FAs can decrease the insulin and the glucose levels in mice via PPARγ activation, as TZD rosiglitazone does, but without the severe side-effects of rosiglitazone, which is mainly gaining weight [62]. The NO_2_-FAs tested in this work formed covalent adducts with Cys285 via a Michael addition. Derivatives of NO_2_-OA were found to be therapeutically efficient against metabolic syndrome in obese zucker rats [66]. Obese zucker rats (ob/ob rats) are mutant mice that eat excessively due to mutations in the gene, which is responsible for the production of leptin, a hormone responsible for the control of the appetite. Zucker rats are a model of type II diabetes. NO_2_-OA derivatives managed to decrease dramatically the food intake of these rats, a state which lasted during the whole experiment period (~14 days) and caused a significant decrease of weight in the obese rats. This treatment also decreased the triglycerides found in plasma and normalized the plasma free FAs.

Non-alcoholic fatty liver disease (NAFLD) is the most dominant disease that the liver suffers. NAFLD is a common disease to almost 90% of obese adults and correlates with diseases like obesity, type 2 diabetes and dyslipidemia. The most common mark that indicates NAFLD is the lipid accumulation inside the liver (steatosis) without consuming significant amount of alcohol. Non-alcoholic steatohepatitis (NASH) can lead to severe diseases (cirrhosis, hepatic carcinoma, liver failure) and finally to death [67]. The significance of the discovery of new potent drugs to overcome the harmful effects of NASH has emerged. Based on results from a series of clinical trials, the most potent drugs against NASH are TZDs as insulin sensitizers [68]. To fight NASH and encounter at the same time the side-effects of TZDs, electrophilic NO_2_-FAs have been found to be efficient agents against NAFLD. Freeman’s work in 2019 shows that the tested electrophilic NO_2_-FAs are promising drugs against NAFLD, without the adverse side-effects of TZDs [69]. ΝO_2_-OA has also contributed significantly to energy metabolism and to the inhibition of hepatic triglyceride accumulation [70]. In general, NO_2_-OA has been reported to benefit the organism to conditions of steatohepatitis and fibrosis.

In chronic kidney disease (CKD), a variety of harmful mechanisms, including oxidant stress and inflammation, correlated with dysregulation of NF-κB pathway and Nrf2 protein. From the other side, some protective mechanisms of the organism, which can be activated by NO_2_-FAs, can decrease these side-effects. In the study of Jorkasky in 2019 [71], 10-NO_2_-OA was evaluated for the treatment of uni-nephrectomized mice that were receiving deoxy-corticosterone acetate (DOCA)-high salt diet. 10-NO_2_-OA increased the heart and kidney weight of the mouse model, as well as it decreased nephrinuria, glomerular hypertrophy and glomerusclerosis. In this study, CXA-10 was compared with enalapril, an anti-hypertensive drug, and it showed some interesting results in which enalapril did not. Glomerular protection, anti-fibrotic and anti-inflammatory effects were observed in the case of CXA-10 but were not observed in the case of enalapril. The endogenous interaction of NO_2_-FAs with PPARs, whose role is known on the metabolism of glucose and lipids [72], may be the reason for all these beneficial effects of CXA-10 against CKD. Freeman and coworkers have reviewed the beneficial effects of NO_2_-FAs and other electrophilic compounds against several inflammatory and fibrotic diseases, discussing their characteristics and their pharmacology [73].

In vascular diseases—for example, ischemia-reperfusion injury (IRI or I/R)—it is of importance to regulate apoptosis. Nie and coworkers have demonstrated that CXA-10 is able to weaken OGD/R-induced apoptosis [72]. This effect was achieved by inhibition of Bax translocation and activation and an upcoming mitochondrial apoptotic cascade, and it was proved to be PPARγ-dependent.

**Figure 7:**
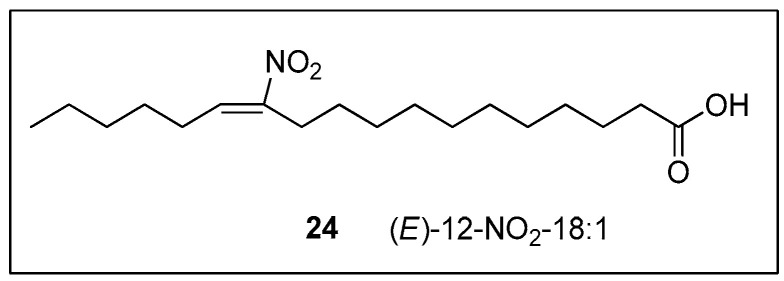
Structure of the optimum activator of PPARγ reported by Gorczynski [74].

In 2009, Gorczynski and coworkers reported the optimum structural features of NO_2_-FAs as PPARγ agonists, in order to activate PPARγ and exhibit all its beneficial effects, ready to decrease or remove possible inflammation or oxidation stress [74]. The main result of this work is that a 12-nitrated position of the unsaturated acid is essential for the optimum activation of PPARγ. Some NO_2_-FAs exhibited better ligand affinity than rosiglitazone [75]. Table 1 summarizes the IC_50_ values of various NO_2_-FAs measured by a scintillation proximity competitive binding assay. The structure of the optimum agonist, which induces the activation of PPARγ, is shown in Figure 7.

The importance of understanding the mechanism by which PPARγ is activated by agonists is of great importance for encountering inflammation and many other PPARγ-dependent systems. For example, PPARγ is reported to inhibit NF-κB dependent transcriptional activation in skeletal muscle [76]. The transcriptional activation of NF-κB in skeletal muscle is associated with skeletal muscle atrophy and insulin resistance and can be caused due to long-term NF-κB activation [77,78,79]. Thus, NO_2_-FAs could be alternative therapeutic agents for this type of disease. Administration of NO_2_-OA has also been shown to be beneficial against Abdominal Aortic Aneurysm (AAA), which is one of the diseases with the highest frequency of death globally [80].

In 2002, Karin and coworkers reviewed a correlation between NF-κB signaling pathway and cancer [81]. Disruption of the physiological regulation of NF-κB by the IKK protein complex (four subunits: IKKα, IKKβ, IKKγ, IKKδ) leads to NF-κB’s activity, which causes the transcription of inflammatory-genes, resulting in the generation of many inflammatory mediators. The NF-κB-induced triple negative breast cancer was also reported previously by Woodcock et al. [31]. PPARγ, as a modulator of cellular differentiation and regulator of tumorigenesis, was found to be expressed in many different types of cancer cells and its activation by agonists leads to either cell proliferation inhibition or cancel cell apoptosis [82,83]. The expression of PPARγ in many different types of cancer shows the complexity and the diversity of the biological action of how nitroalkenes overall attenuate inflammation and cancer [84].

### 2.4. NO_2_-FAs as Regulators of Miscellaneous Targets

NO_2_-FAs have been found to be responsible for the treatment of vascular endothelial cells, resulting in a dose-dependent upregulation of correlated HSPs (HSPA6, HPA1A, DNAJA4, HSPB8) by the expression of Heat Shock Factor 1 and 2 (HSF-1 and HSF-2) [15,85]. Heat Shock Proteins (HSPs) are a family of proteins, which are generated by cells under stress conditions. First, they were described as proteins associated only with heat shock [86], but several reports show a correlation with cold, UV light, and with processes of healing or tissue regeneration [87,88,89].

Another interesting bioactivity of NO_2_-FAs, demonstrated in a murine model, is the inhibition of sEH with the same way presented before, by forming covalent adducts with Cys521 via a Michael addition to the electrophilic double bond [90]. Administration of these electrophilic compounds results in the reduction of blood pressure to mice suffering from hypertension. Inhibition of sEH prevents hydrolysis of the epoxyeicosatrienoic acid substrates, so their accumulation induces vasodilation lowering blood pressure.

Xanthine oxidoreductase (XOR) is a molybdoflavin protein responsible for the oxidation of hypoxanthine to xanthine and finally to uric acid, resulting in the reduction of NAD^+^ to NADH [91]. XOR is the enzyme responsible for the regulation of the final process of purine catabolism and mainly generates oxidants responsible for the generation of inflammation and secondary species of nitrogen [92,93,94]. NO_2_-OA inhibits XOR with a low IC_50_ value (0.6 μM) and decreases significantly the generation of superoxide O_2_^−^. Superoxide is one of the Reactive Oxygen Species (ROS), which in oxidative stress its formation grows and causes harmful effects to proteins, lipids and nucleic acids [95].

As reported by Hansen et al. in 2018, NO_2_-FAs can be formed in response to virus infection and are able to inhibit stimulator of interferon genes (STING) signaling, leading this way to an anti-inflammatory activity against several diseases, such as, systemic lupus erythematosus, Aicardi-Goutiéres syndrome and STING-associated vasculopathy [96]. With this work, 9-NO_2_-OA, 10-NO_2_-OA and NO_2_-cLA are shown to be capable of targeting STING signaling and reduce the release of type I IFNs in murine and human cells. Thus, these endogenously formed nitrated compounds can overcome STING-induced inflammation.

**Figure 8:**
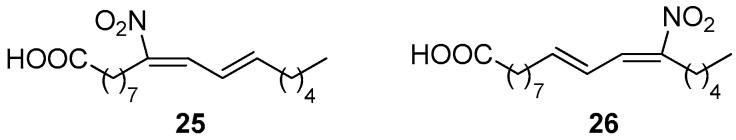
Structures of the NO_2_-FAs reported by Denicola and coworkers, which act as activators of SIRT6 [97].

NO_2_-FAs have also been demonstrated to be activators of Sirtuin 6 (SIRT6), a protein crucial for both glucose and lipid homeostasis, which is found to be actively involved in maintaining the DNA of the organism stable [97]. In this work, a SAR study using 9-NO_2_-OA (**9**), 10-NO_2_-OA (**1**), 9-NO_2_-cLA (**25**) and 12-NO_2_-cLA (**26**) (Figure 8) showed that these NO_2_-FAs are significant in vitro activators of SIRT6. The formation of a covalent adduct between NO_2_-FA and Cys18 of SIRT6, caused a change to the conformation of the protein, inducing this way a greater activation of SIRT6 (40-fold at 20 μΜ). This Cys18 residue is present only at the *N* terminus of SIRT6 and absent to other isoforms of the protein, making this the only way SIRT6 is able to react with these electrophilic compounds.

Pereckova and coworkers recently have shown that NO_2_-OA is involved in the regulation of stem cell pluripotency and differentiation of mouse Embryonic Stem Cells (mESC) via signal transducer and activator of transcription 3 (STAT3) signaling [98]. NO_2_-OA was found to modulate pluripotency of mESC via the regulation of STAT3 phosphorylation, as well as to attenuate cardiac differentiation and to guide mESC into neural fate.

A recent report by Mathers and coworkers demonstrated the effect of NO_2_-FAs on psoriasiform dermatitis [99]. Several murine models of psoriasis were employed in order to study the anti-inflammatory activity of NO_2_-OA. Oral administration of NO_2_-OA downregulated the formation of inflammatory cytokines in the skin, and in vitro experiments showed a decrease of the formation of basal IL-6 levels and IL-17A-induced expression of IL-6, via the NF-κB signaling pathway. Furthermore, NO_2_-OA downregulated the phosphorylation and nuclear translocation of STAT3 by nitroalkylating STAT3 and inhibiting this way the keratinocyte proliferation. By blocking NF-κB and STAT3, NO_2_-FAs may constitute new therapeutic agents to treat psoriasis.

A mammalian enzyme—namely, Prostaglandin Reductase-1 (PtGR-1), able to metabolize NO_2_-FAs in vivo—has been identified [100]. PtGR-1 reduces fatty acid nitroalkenes to nitroalkanes, deactivating them and silencing their signaling reactions.

## 3. Syntheses of NO_2_-FAs

**Scheme 3:**
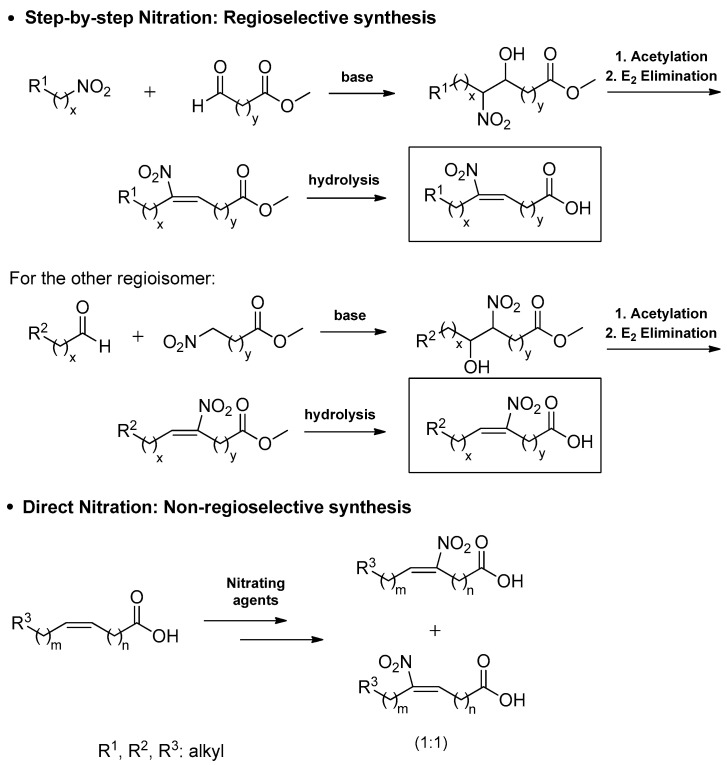
Different general strategies for the synthesis of NO_2_-FAs.

As described before in Scheme 2, the parent mono- or poly-unsaturated compounds can be nitrated endogenously by the reactive species of nitrogen monoxide and generate nitroalkenes [1,2,3]. However, the importance of NO_2_-FAs and their potential applications to treat inflammatory diseases or cancer has led to the development of various strategies for their synthesis in the laboratory. The different routes, which involve either direct and non-selective nitration or step-by-step and regioselective nitration, are summarized in Scheme 3.

### 3.1. Direct Nitration of Unsaturated Lipids Yielding Derivatives of NO_2_-FAs

**Scheme 4:**
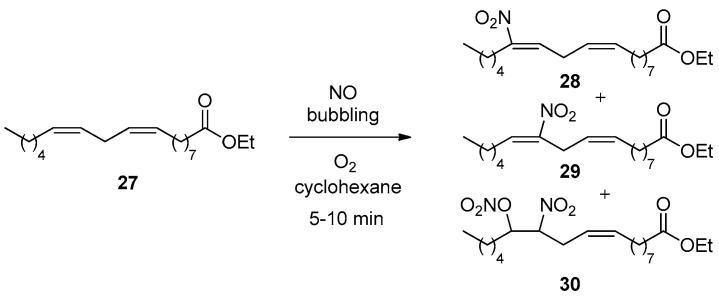
Synthesis of nitro derivatives of ethyl linoleate.

The first direct nitration of LA or its ethyl ester by exposure to nitrogen monoxide and oxygen in cyclohexane was reported in 1996 by D’Ischia (Scheme 4) [101].

**Figure 9:**
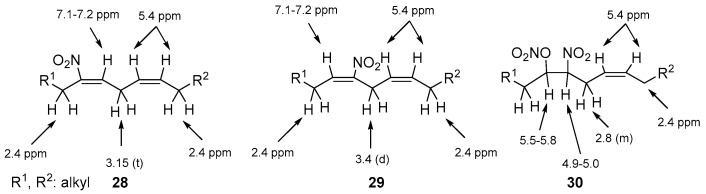
Gas-induced synthesis of nitro isomers and some of their characteristic ^1^H-NMR shifts [101].

GC-MS and NMR studies showed the synthesis of an inseparable mixture containing three isomeric compounds: nitroalkenes **28**, **29** and nitronitrate derivative **30** (Scheme 4). The structure of these formed nitro derivatives was based on the characteristic for each isomer ^1^H-NMR data (Figure 9). This mixture of isomers started probably by a first addition of NO_2_ to one of the double bonds of ethyl linoleate, formed by a possible autoxidation of NO [102].

**Scheme 5:**
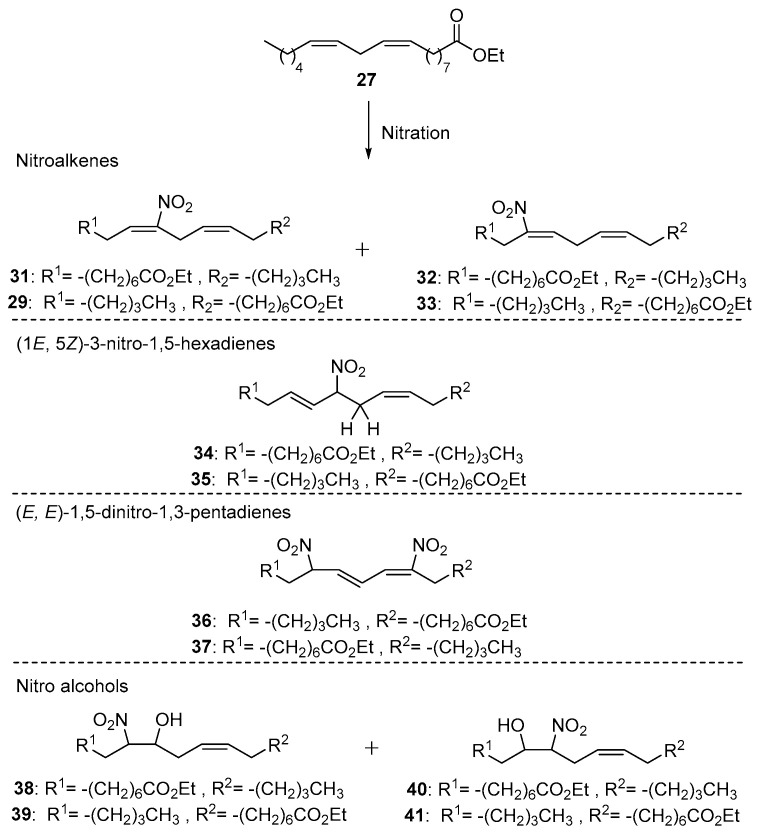
Ethyl linoleate and its nitro derivatives including nitroalkenes **29**, **31**–**33**.

In 2000, D’Ischia studied the possible nitro products and by-products derived by the nitration of ethyl linoleate (**27**), yielding different isomers of nitroalkenes (Scheme 5).

**Scheme 6:**
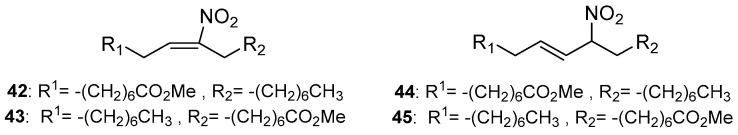
Nitroalkenes and by-products formed by nitration of methyl oleate.

The reactivity of this unsaturated ester over different nitrating conditions was extensively described; also, the NMR data of the different formed nitro isomers were recorded [103]. The employed nitrating agents in this work were nitronium tetrafluoroborate (NO_2_BF_4_), NO or a NO_2_^−^ solution. Different regio-isomers of nitroalkenes were formed (compounds **29**, **31**–**33**), as well as different types of nitro by-products like: 3-nitro-1,5-hexadienes (**34** and **35**), 1,5-dinitro-1,3-pentadienes (**36** and **37**) and four different regio-isomers of nitro alcohols **38**–**41**. In other experiments D’Ischia studied the monounsaturated fatty ester methyl oleate for its nitration reaction properties (Scheme 6). Mainly, a ratio 1:1 of (**42** and **43**):(**44** and **45**) was observed by the same nitration procedures followed for ethyl linoleate.

**Scheme 7:**
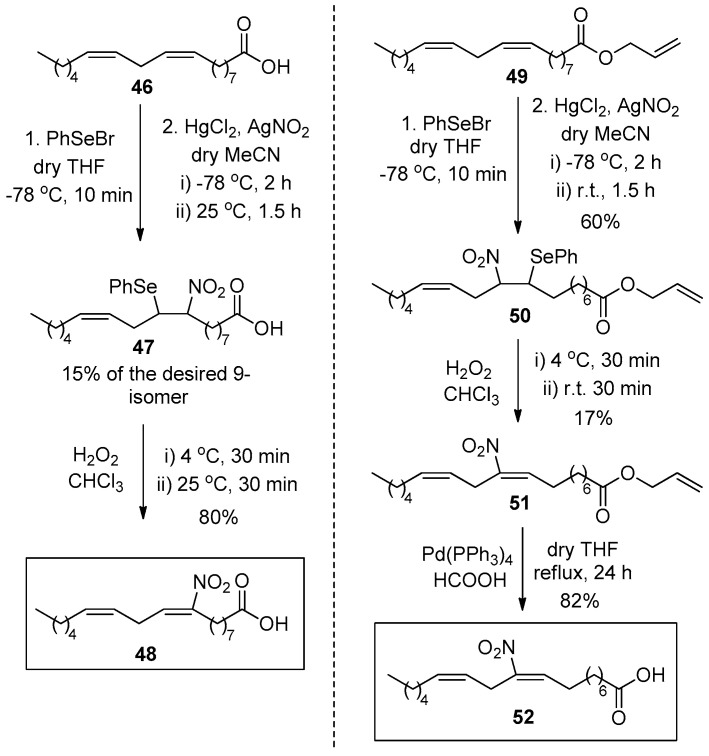
Synthesis of 9- and 10-NO_2_-LA using selenium-based method [104].

In 2008, D’Ischia reported a way to synthesize 9- and 10-NO_2_-LA, **48** and **52** respectively, by employing PhSeBr, HgCl_2_ and AgNO_2_ in dry MeCN, followed by the treatment of H_2_O_2_ in chloroform (Scheme 7) [104].

The synthesis of 10-NO_2_-LA is similar to that applied for the 9-nitro regio-isomer by only changing the free carboxyl group to an allyl ester, which gets cleaved in a final step by Pd(PPh_3_)_4_ and HCOOH in dry tetrahydrofuran (THF). During these studies, three decomposition products of 9-NO_2_-LA were observed, after incubation in a phosphate buffer of pH 7.4.

**Scheme 8:**
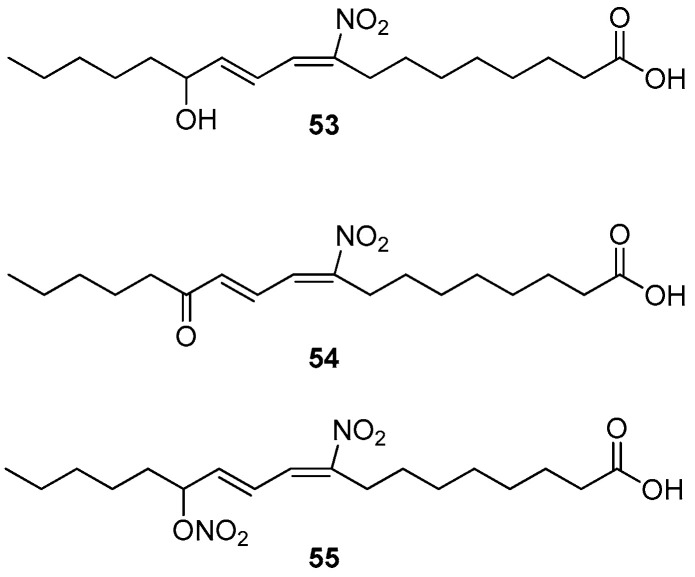
Degradation products after incubation of 9-NO_2_-LA at pH 7.4.

All three by-products were UV-Vis active in the TLC and were positive to Griess reagent, indicating the presence of a nitro group. By-products **53**–**55** are shown in Scheme 8.

**Scheme 9:**
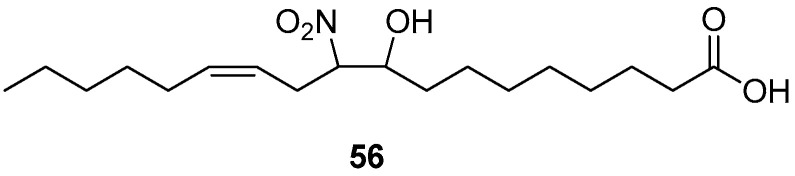
Unique decay product of 10-NO_2_-LA when exposed to pH 7.4.

When 10-NO_2_-LA **52** was exposed to aqueous phosphate buffer pH 7.4, only one decay product was observed. (12*Z*)-9-Hydroxy-10-nitrooctadec-12-enoic acid (**56**) was fully characterized by 1D and 2D NMR experiments and its structure is shown below in Scheme 9.

**Scheme 10:**
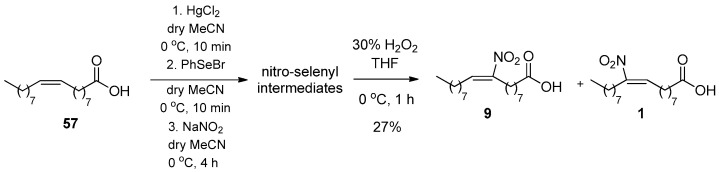
Synthesis of a mixture of 9- and 10-NO_2_-OA by the nitro-selenylation intermediate method [105].

In 2013, Schopfer reported the synthesis of a mixture of 9- and 10-NO_2_-OA (**9** and **1**, respectively) following a modification of the method described by D’Ischia in 2008, where NaNO_2_ was used instead of AgNO_2_ (Scheme 10) [105]. After the consecutive additions for the formation of nitro-selenyl intermediates, the oxidative cleavage of PhSe-group takes place with 30% H_2_O_2_ in THF for 1 h.

**Scheme 11:**
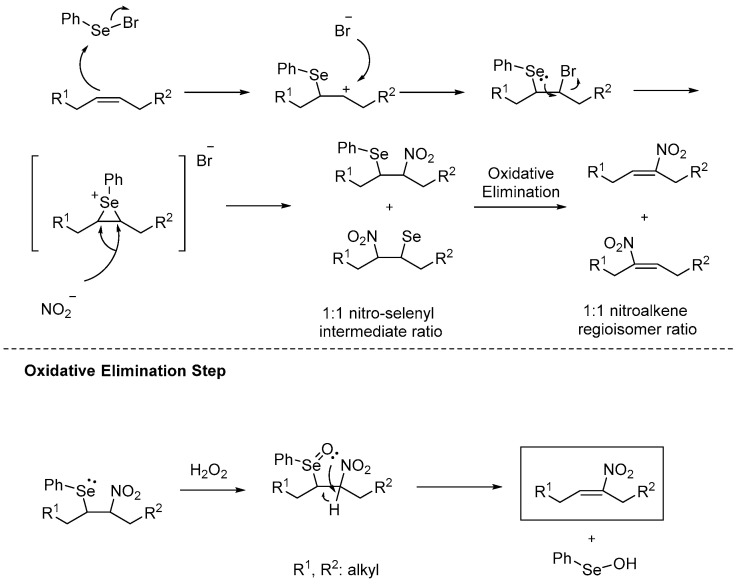
Proposed mechanism of the synthesis of 9- and 10-NO_2_-OA via nitro-selenylation, followed by oxidative elimination.

The reaction mechanism (Scheme 11) begins with the nucleophilic attack of the double bond to the selenium atom, which affords the vicinal bromoselenium [106,107]. After intramolecular nucleophilic attack, a three membered ring, including the positively charged selenium atom, occurs.

Now the nitrating source (AgNO_2_ or NaNO_2_) has 1:1 chance to attack the two electrophilic carbon atoms of the three membered ring, resulting in a 1:1 regio-isomer ratio of the formed nitro-selenyl intermediates. The nitro-selenyl compound reacts with hydrogen peroxide to give a compound bearing an oxidized form of selenium, which can now undergo an intramolecular elimination, affording phenylselanol and the desired nitroalkene.

In 2021, Manolikakes and coworkers published a review article summarizing different nitration procedures of alkenes [108]. In this review, different nitrating and oxidizing agents are described, for the direct nitration of an alkene leading finally to the nitroalkenyl moiety, which constitutes the reactive electrophilic moiety of NO_2_-FAs.

### 3.2. Step-By-Step Synthesis of NO_2_-FAs

**Scheme 12:**
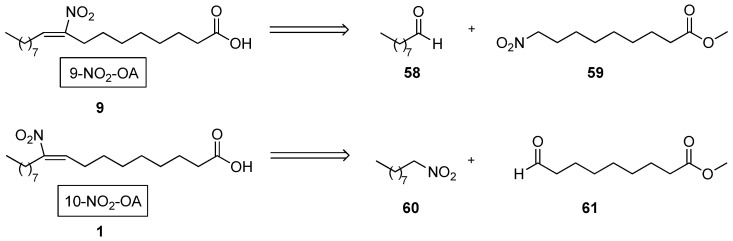
Retrosynthetic analysis for 9- and 10-NO_2_-OA.

In 2006, Gorczynski and coworkers reported regio and stereospecific syntheses of (*E*)-9-NO_2_-OA and (*E*)-10-NO_2_-OA (**9** and **1**) in eight and nine steps, respectively [94,109]. The retrosynthetic analysis (Scheme 12) of these nitroalkenes showcases the need for the corresponding nitroalkanes or aldehydes, depending on the desired position of the nitro group on the double bond.

**Scheme 13:**
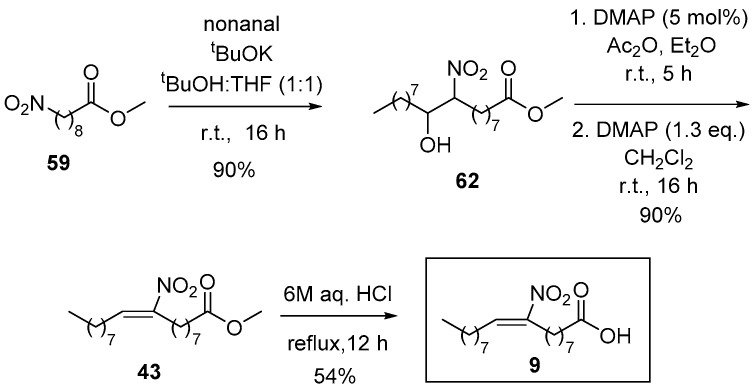
Regiospecific synthesis of 9-NO_2_-OA by Gorczynski [109].

For the synthesis of 9-NO_2_-OA (Scheme 13), methyl 9-nitrononanoate (**59**) was synthesized in three steps by a Henry reaction between nitromethane and the corresponding aldehyde, an *O*-acetylation and a reduction using NaBH_4_ and MeOH (overall yield 74%). Compound **59** was used for a Henry reaction with nonanal under basic conditions. The isolated nitro hydroxy compound **62** was acetylated with acetic anhydride (Ac_2_O) and 4-dimethylaminopyridine (DMAP) in a first step and treatment with excess of DMAP led to the formation of the nitroalkene moiety. The final step was the acidic hydrolysis of the methyl ester to obtain 9-NO_2_-OA (**9**).

**Scheme 14:**
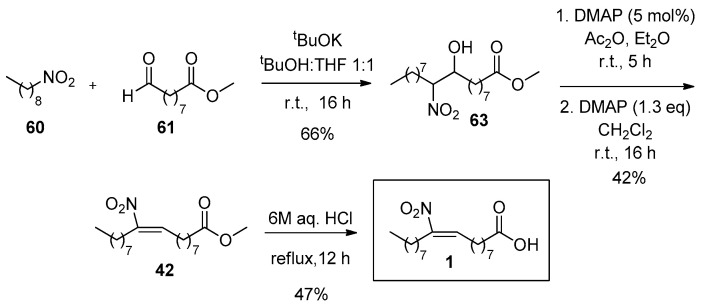
Synthetic route for 10-NO_2_-OA [109].

The synthesis of 10-NO_2_-OA (**1**) is similar with the synthetic pathway presented for the 9-regio-isomer. The required precursor compounds for the Henry reaction are 1-nitrononane (**60**) and methyl 9-oxononanoate (**61**) (Scheme 14). Compound **60** can be synthesized from the corresponding precursor aldehyde reacting with nitromethane to give the nitro hydroxy compound and then a subsequent reduction with NaBH_4_ affords 1-nitrononane.

As described in Gorczynski’s work, 9-oxononanoate (**61**) can be synthesized in three steps; a reduction of monomethyl azelaic acid with BH_3_-THF and a PCC oxidation affords one of the starting materials for the Henry reaction [109]. The reported yields for this NO_2_-FA at the late stages of the synthesis are slightly decreased compared with 9-NO_2_-OA.

**Scheme 15:**
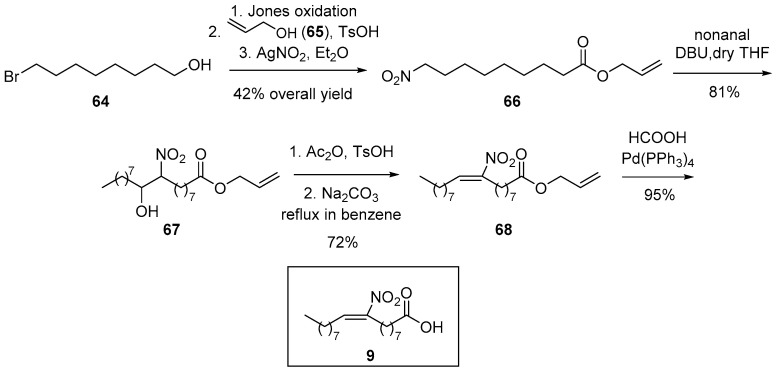
Synthesis of 9-NO_2_-OA by Branchaud’s protocol [110].

In the same year, Branchaud and coworkers reported useful synthetic routes affording all four isomers of nitro oleic acids: (*E*), (*Z*), 9- and 10-NO_2_-OA [110]. The synthesis of (*E*)-9-NO_2_-OA started from 8-bromononan-1-ol (**64**), with a Jones oxidation, an esterification with allyl alcohol (**65**) and a nitration with AgNO_2_ in diethyl ether (Scheme 15). The synthesized nitro allyl ester **66** reacted with nonanal and 1,8-diazabicyclo(5.4.0)undec-7-ene (DBU) to give the nitro hydroxy ester **67** in a good yield. An acid-catalyzed acetylation using Ac_2_O and p-toluenesulfonic acid (TsOH) gave the nitro acetate compound, which under basic conditions and reflux in benzene afforded nitroalkene **68**. A final cleavage of the allyl ester using Pd(PPh_3_)_4_ and HCOOH in THF gave 9-NO_2_-OA.

**Scheme 16:**
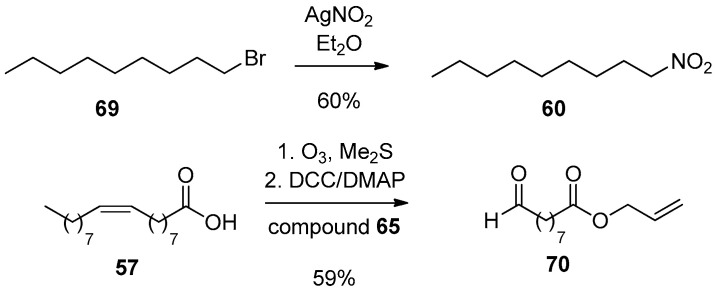
Synthesis of the precursor compounds for 10-NO_2_-OA [110].

The needed precursors for the synthesis of 10-NO_2_-OA were synthesized as shown in Scheme 16. Nitration of 1-bromononane (**69**) with AgNO_2_ in diethyl ether gave compound **60**. The needed aldehyde **70** was synthesized by ozonolysis of OA (**57**), followed by reductive workup with Me_2_S, resulting in a mixture of aldehydes. 9-Oxononanoic acid was isolated and its Steglich esterification with allyl alcohol using *N*,*N*’-dicyclohexylcarbodiimide (DCC)/DMAP afforded the desired allyl 9-oxononanoate.

**Scheme 17:**
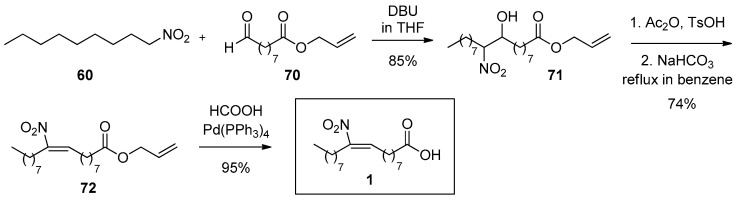
Synthesis of 10-NO_2_-OA by Branchaud’s protocol [110].

A Henry condensation of **60** with **70** afforded the nitro hydroxy product **71** in 85% yield (Scheme 17). The three final steps of the synthesis are the same as described before with similar yields (85%, 74% and 95% respectively).

In this work, all four possible isomers, (*E*), (*Z*), 9-, 10- of NO_2_-OA were synthesized, isolated and fully characterized by NMR [110]. The NMR data showed that the (*E*) nitroalkene proton is shifted at 7.10 ppm, unlike the (*Ζ*) shift that appears at 5.70 ppm, in both 9- and 10-NO_2_-OA. These NMR shifts can be used as a diagnostic tool.

**Scheme 18:**
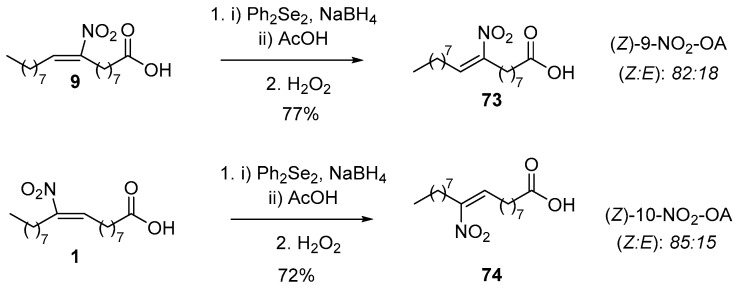
Isomerization of (*E*) nitroalkenes to (*Z*) [110].

Branchaud’s protocol has the capability of forming (*Z*) nitroalkenes from their (*E*) isomers by first employing Ph_2_Se_2_, NaBH_4_ and acetic acid (AcOH), and in a second step H_2_O_2_, affording this way any isomer of the desired nitroalkenes (Scheme 18). The isomer ratio of the observed *Z*:*E* nitroalkenes was calculated by ^1^H-NMR. For compound **73**, the isomer ratio was found to be 82:18 and for compound **74** was 85:15.

In 2008, Pellacani, et al. described a facile and highly stereoselective one-pot synthesis of either (*E*)- or (*Z*)-nitro alkenes by simply changing solvent and temperature [111]. Several aldehydes and nitroalkanes were combined to give either (*E*) or (*Z*) nitroalkenes by the employment of piperidine and molecular sieves. The first method employed CH_2_Cl_2_ at r. t. for 30 min and the second method utilized toluene under reflux for 4 h. This methodology was applied only for the formation of simple molecules bearing a nitroalkene moiety without any further functional groups. For example, propanal, pentanal, hexanal and isobutanal were used for a one-pot Henry condensation reaction with nitroethane or 1-nitropropane to afford either (*E*) or (*Z*) nitroalkenes.

**Scheme 19:**
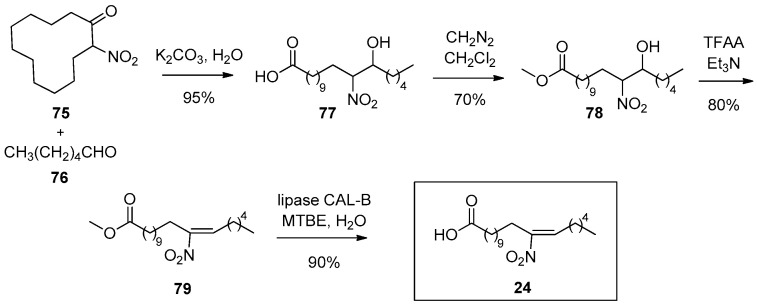
Zanoni’s methodology affording nitroalkene **24** in 50% overall yield [112].

In 2010, Zanoni reported a much more efficient synthetic route to (*E*)-12-nitrooctadec-12-enoic acid (**24**) in 4 steps (overall yield 50%), outperforming the previously reported methodology of Gorczynski [70], which had a 4.8% overall yield (Scheme 19) [112]. This high-yield synthesis of nitroalkene **24** began with a one-pot consecutive Henry reaction of 2-nitrocyclododecanone (**75**) with hexanal (**76**), followed by a retro-Claisen ring cleavage, affording this way the resulting nitro hydroxy acid **77**. Esterification with diazomethane (CH_2_NH_2_) in CH_2_Cl_2_ afforded methyl ester **78**, which, after dehydration by treatment with trifluoroacetic anhydride (TFAA), led to nitroalkene **79**. In this final step of synthesis, Zanoni employed Candida Antartica lipase B (CAL-B) in order to smoothly hydrolyze the methyl ester and avoid the use of harsh conditions, as Gorczynski does. CAL-B is an insensitive pH enzyme, so it can cleave the methyl ester in an organic solvent, like methyl *tert*-butyl ether (MTBE) [113]. A minimum amount of water exists in the reaction, in order to cleave the intermediate acyl enzyme [114,115].

**Scheme 20:**
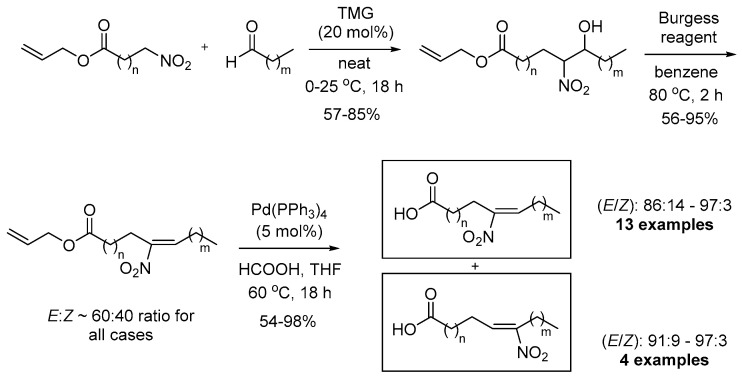
Manolikakes’ developed protocol for the synthesis of different NO_2_-FAs [116].

In 2016, Manolikakes and his coworkers presented the synthesis of a plethora of NO_2_-FAs [116]. After the preparation of the required starting compounds with either a Victor Meyer reaction or a Kornblum oxidation, the synthetic route began with a nitroaldol reaction between the nitroalkane and the used aldehyde each time, using a catalytic amount of 1,1,3,3-tetramethylguanidine (TMG) as the base (Scheme 20). The dehydration process was promoted by the Burgess reagent in benzene at 80 °C for 2 h, followed by a final deprotection of the allyl ester for the formation of the final NO*_2_*-FA. The *E*:*Z* 60:40 isomeric ratio for almost all examples turned into greater than 86:14, due to the isomerization of the double bond by the employed Pd(PPh_3_)_4_, HCOOH system.

This final isomerization afforded products with a high selectivity of *E* isomer over *Z*, providing to the method great selectivity with facile means. In total, seventeen different NO_2_-FAs, thirteen with the nitro group located close to the carboxyl group, and four with the nitro group far from it, were synthesized and their biological activity was left to be discovered.

**Scheme 21:**
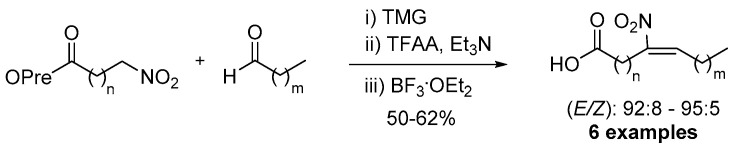
One-pot transformation for the selective formation of (*E*) NO_2_-FAs by Manolikakes [117].

Very recently Manolikakes and coworkers reported the one-pot synthesis of NO_2_-FAs by modifying their pre-existant synthetic methodology to a quicker and easier to use protocol (Scheme 21) [117]. In this methodology, prenyl esters and aldehydes were used as starting materials by using (i) TMG, (ii) TFAA, Et_3_N and (iii) BF_3_**^.^**OEt_2_ and affording the desired (*E*) NO_2_-FAs.

**Scheme 22:**
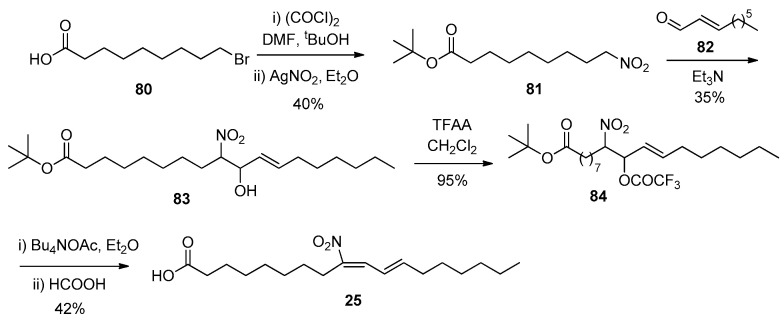
Synthesis of 9-NO_2_-cLA [118].

**Scheme 23:**
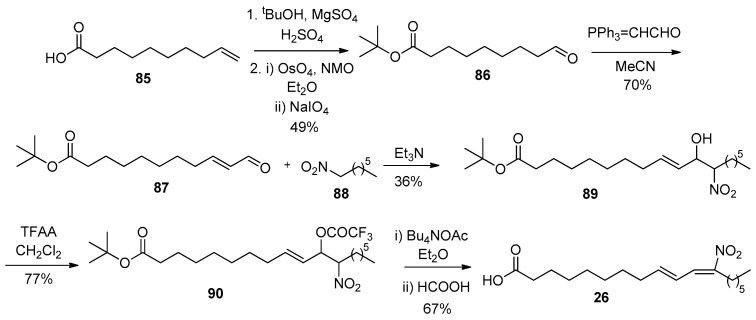
Synthesis of 12-NO_2_-cLA [118].

**Scheme 24:**
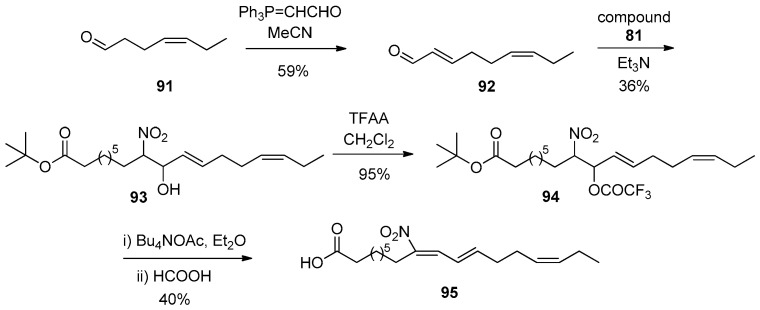
Synthesis of 9-nitrorumelenic acid [118].

In 2021, Freeman reported the synthesis of 9- and 12-NO_2_-LA, as well as 9-nitrorumelenic acid (Scheme 22, Scheme 23 and Scheme 24) [118]. For the synthesis of 9-NO_2_-LA, starting from ω-bromo carboxylic acid **80** by employing oxalyl chloride (COCl)_2_, *N*,*N*-dimethylformamide (DMF) in ^t^BuOH and then AgNO_2_ in Et_2_O, nitro compound **81** was obtained. Then, by employing non-2-enal (**82**) and Et_3_N, nitro hydroxy derivative **83** was obtained in 35% yield, which then reacted with TFAA, affording compound **84**. Treatment of compound **84**, first with Bu_4_NOAc and then with HCOOH, afforded 9-NO_2_-cLA (***25***) in 42% yield. For the synthesis of 12-NO_2_-LA, ω-UFA **85** was converted into the corresponding *tert*-butyl ester, and then treated with osmium tetroxide, *N*-methyl morpholine *N*-oxide (NMO), and NaIO_4_, to give aldehyde **86**. After a Wittig and a Henry reaction, nitro hydroxy compound **89** was treated with TFAA to afford compound **90**. Treatment with Bu_4_NOAc in Et_2_O and finally with formic acid afforded 12-NO_2_-cLA (**26**). 9-Nitrorumelenic acid (**96**) was successfully synthesized following similar synthetic steps.

**Scheme 25:**
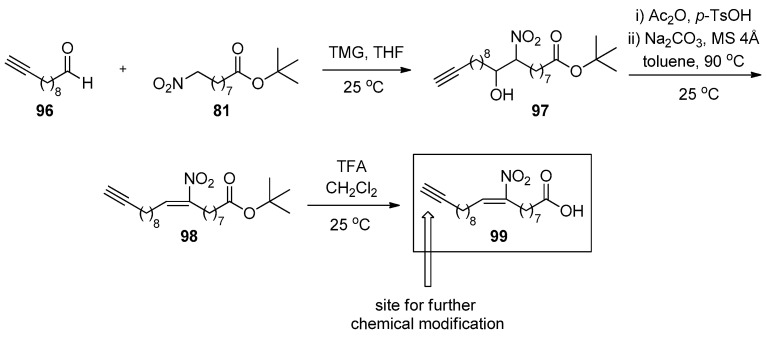
Synthesis of 9-nitroeicos-9-en-19-ynoic acid (**99**) [119].

In 2021, Zhang et al. reported the synthesis of 9-nitroeicos-9-en-19-ynoic acid (**99**), a derivative of 9-NO_2_-OA, where a terminal triple bond exists (Scheme 25) [119]. Compound **99** may serve as a tool of detecting in general which mammalian proteins can form covalent adducts with the electrophilic center of this NO_2_-FA. This accessible triple bond suggests a key to access multiple derivatives of the corresponding NO_2_-FA. The organic base used for the Henry reaction is TMG and the formation of the nitroalkene moiety started by using Ac_2_O and TsOH. After the initial acetylation reaction was over, Na_2_CO_3_ was used in toluene at 90 °C, in order to form the nitrated double bond.

## 4. Conclusions

NO_2_-FAs constitute a class of anti-inflammatory agents acting through the interaction with various important proteins, such as Keap-1, Nrf2, NF-κB, HSPs or the PPARγ receptor. Their main mode of interaction is by forming covalent adducts with cysteine residues of important biological targets via a reversible Michael addition. NO_2_-FAs can be endogenously synthesized by the reaction of UFAs with reactive species of nitrogen monoxide. Despite the endogenous nitration of UFAs, NO_2_-FAs may be obtained by the direct nitration of UFAs with several nitrating agents, mainly affording mixtures of the corresponding nitro regio-isomers. Another efficient way to obtain NO_2_-FAs, where the nitro group now has a specific position at the double bond, is by following a step-by-step strategic synthesis, starting from the required aldehydes and nitroalkanes. Efficient one-pot syntheses of NO_2_-FAs have been developed as well, introducing in this way an easier to use synthetic procedure for the synthesis of a variety of NO_2_-FAs.

## Figures and Tables

**Table 1:** NO_2_-FA-PPAR SAR study. *E*-12-NO_2_-18:1 exhibits the highest activity as PPARγ agonist.

Compound	IC_50_ (μM)		Ref.
	PPARγ	PPARα	
Rosiglitazone	0.25	>15	[75]
*E*-12-NO_2_-18:2	0.41	9.6	[75]
*E*-9-NO_2_-18:1	0.98	>15	[74]
*E*-10-NO_2_-18:1	1.61	>15	[74]
*Z*-9/10-NO_2_-18:1	1.73	10.8	[74]
*E*-9-NO_2_-16:1	0.83	>15	[74]
*E*-10-NO_2_-16:1	0.63	>15	[74]
*E*-5-NO_2_-18:1	1.68	>15	[74]
*E*-6-NO_2_-18:1	1.72	>15	[74]
*E*-12-NO_2_-18:1	0.039	1.6	[74]
*E*-13-NO_2_-18:1	0.19	1.1	[74]

## Data Availability

Not applicable.
